# New energy vehicles and the political geoecology of China’s Ecological Civilisation

**DOI:** 10.1177/23996544241231683

**Published:** 2024-02-05

**Authors:** Pablo I Ampuero-Ruiz

**Affiliations:** 1234 Universiteit van Amsterdam, The Netherlands

**Keywords:** China, Ecological Civilisation, new energy vehicles, political geoecology, energy transition

## Abstract

Ecological Civilisation has been China’s policy concept to frame its environmental sustainability strategy. While it lacks a clear definition, its multiple practices show a reliance on the development and implementation of technologies that reduce pollution levels and beautify the country. One of those technologies are New Energy Vehicles (NEV), which stand at the centre of an e-mobility transition across urban China. I engage with the growing debate on China’s Ecological Civilisation and e-mobility transition by reflecting on the critical junctions between this policy concept and the NEV industry. I suggest that the New Energy requirements of China’s Ecological Civilisation rely on power relations that enhance state capacity domestically and transnationally. In this sense, the pursuit of New Energy to build China’s Ecological Civilisation relies on moral and sovereign forms of power. In a context of increasing tensions between China and countries in the North Atlantic, my contribution shows that these forms of power enhance China’s state capacity through its economic infrastructure, re-producing old dependencies and inequalities.

## Introduction

*China 2050* (Hu et al., 2018) is a book that imagines how the People’s Republic of China (PRC) would look like once its socialist modernisation (*shehui zhuyi xiandaihua* 社会主义现代化) is completed. Edited by Hu An’gang—a well-known intellectual of China’s New Left affiliated with Tsinghua University and a recurrent commentator on the “Chinese Model”—the book staunchly defends the country’s path to development. The basic premise of the book is that only by pursuing a path of socialist modernisation can the Chinese Dream (*zhongguo meng* 中国梦) be achieved (2018: 3)^1^. This dream encapsulates the challenges of providing happier and better lives to its people while juggling the historical legacies of an ancient civilisation, a powerful Eastern country with a massive population, and a former world power that was once the most prosperous and has recently emerged from a period of backwardness and weakness (2018: 3). In the more specific chapter presenting the general layout of modernization (*xiandaihua zongti buju* 现代化总体布局), the authors introduce their reflections on Ecological Civilisation (*shengtai wenming* 生态文明); a subsection wedged between society and national security. Here, it briefly acknowledges that “the construction of a Beautiful China […] recognises that protecting the environment is at the same time protecting the productive forces” (2018: 108). This includes a transition from “black” (*heise* 黑色) industrialization, urbanization, and modernization to “green” (*lüse* 绿色) ones through a “period of ecological surplus” (*shengtai yingyu qi* 生态盈余期)—referring to a phase where the growth of green industries unfolds at a higher rate than of black ones—and the realisation of the ancient Chinese idea that “humanity is an integral part of nature” (*tianren heyi* 天人合一) (2018: 109). In these pages, the book condenses some of the core points in the implementation of Ecological Civilisation, namely, that it is possible to secure economic growth while protecting the biodiversity and ecosystems, and that these goals require technological solutions.

In this article, I reflect on the critical junctions affecting the conceptualisation of Ecological Civilisation in the People’s Republic of China. To illustrate the limits to this concept, I build a case around the expansion of the New Energy Vehicles (NEVs) industry, which the PRC government has actively promoted as a technology for reducing urban pollution in Ecological Civilisation national experimental zones (*guojia shengtai wenming shiyanqu* 国家生态文明试验区) (State Council of the People’s Republic of China, 2020).^2^ While the analysis is centred around the flow of resources for Li-ion batteries for Electric Vehicles (EVs), throughout the text I use the Chinese government’s category of New Energy Vehicles, as this is the preferred formulation in their policy framing.

NEVs and EVs are both terms used to describe vehicles that run on electricity, but there are some key differences between them. On the one hand, EVs are powered solely by electricity from batteries, such as in electric cars, buses, or motorcycles. NEVs, on the other hand, include all forms of non-conventional fuel, such as pure electric (*chun diandong qiche* 纯电动汽车), extended range electric vehicles (*zengcheng shi diandong che* 增程式电动车), hybrid electric vehicles (*hunhe dongle qiche* 混合动力汽车), fuel cell electric vehicles (*ranliao dianchi diandong qiche* 燃料电池电动汽车), hydrogen vehicles (*qing fadongji qiche* 氢发动机汽车), among others. Some of these types of NEVs operate in combination with fossil fuels (one is tempted to say “old energy”), such as hybrid EVs, that can change between these fuels, or the extended range EVs that count with a small internal combustion engine (ICE) to provide additional electric power. And in the case of vehicles requiring a charging station, such as pure electric or hybrid electric vehicles, one must consider that coal accounted for 60% of the PRC’s total energy supply in 2019 (International Energy Agency, 2022), which blurs the line between new and old energy sources. In short, in terms of the technology powering the vehicle, all EVs are NEVs, but not all NEVs are EVs.

The NEV industry constitutes an important vantage point into the latest developments of China’s economy. On the one hand, because it is considered an essential industry in the country’s dual circulation strategy (*guonei guoji shuang xunhuan* 国内国际双循环), a plan to simultaneously promote domestic consumption and international trade and investment. And on the other, because it is one of the main industries involved in building an Ecological Civilisation. This makes NEVs a key materiality through which to critically think about the social and socio-technical transformations taking place in a country like China in the context of global energy transitions. The simultaneity of local and transnational transformations is highly relevant to political geoecology analysis, specially when technological fixes, like NEVs for example, overlap with transnational flows of capital and resources associated with the Belt and Road Initiative (*yidai yilu* 一带一路). In this sense, my invitation is to tell a story of domestic and transnational power relations and socioenvironmental tensions emerging from the materiality of New Energy Vehicles.

It has previously been observed that PRC is undergoing a “socio-technical regime” of e-mobility transition, where political and cultural entanglements affect the transformation of the automobility system, making the disruptive potential of electric cars to stall (Tyfield, 2014: 593–594). In recent years, the global sales of electric cars have grown exponentially, but the premises of car ownership and usage have remained unaltered since the expansion of automobility in the 20^th^ century. In this sense, as David Tyfield notes, the expansion of Chinese NEVs could become “a major ‘vehicle’ for both the discursive-political and the material-technological spread of its national hegemony” (2014: 596). To further explore this dimension, I build on Simon Dalby’s “political geoecology” concept, by analysing how power relations “shape particular landscapes, and how more sustainable futures might be constructed in particular places” (Dalby, 2020: 184). In the context of a race for resources for NEVs (Blas and Farchy, 2021: 319; LeVine, 2016; Sanderson, 2022), I seek to engage with ongoing debates about China’s Ecological Civilisation and its transnational dynamics by asking what type of (new) energy is powering the building of this Ecological Civilisation and which forms of power it enables.

Amidst a global race to go green (Sanderson, 2022), it is increasingly relevant to understand the cultural and political realities behind the policies aiming at fostering an energy transition. In the case of the PRC, Ecological Civilisation has become one of the central concepts in its environmental and industrial governance. It stands as the country’s response to climate change in the framing of the United Nations sustainability goals, by mobilising China’s comparative advantages in capital, technology, and knowledge production (Geall and Ely, 2018: 1190–1191). In its political praxis, as Tyfield (2014: 596) observes for the case of electromobility, government environmental action affects power relations by enhancing the state capacity vis-à-vis other actors. These practices resonate with Li Yifei and Judith Shapiro’s (2020) “environmental authoritarianism” trope, referring to the instrumentalization of environmentalism for authoritarian ends in the PRC (2020: 24). In terms of the policy making process, thinking through environmental authoritarianism sets the tone for the analysis of a political reality where rulers establish the meaning of their terms with few, if any, considerations from other actors (Li and Shapiro, 2020: 12). I argue that these underlying power relations are relevant to understand how China’s drive for New Energy affects the development of its NEV industry—by promoting the vertical integration of its production—and the landscapes of countries like Chile, the DRC or Indonesia, whose exports of lithium, cobalt, and other battery materials are central to fuel the construction of an Ecological Civilisation.

The main issues addressed in this paper are: (a) the semantics of Ecological Civilisation in Chinese political discourse, (b) the role of the NEV industry in building an Ecological Civilisation, and (c) the forms of power enabled by the pursuit of New Energy in and from China. In a nutshell, I suggest that the New Energy requirements of China’s Ecological Civilisation rely on power relations that enhance state capacity domestically and transnationally.

## Conceptual genealogies

A growing sense of emergency has engulfed the environmental debates at both local and global instances, albeit with different levels of engagement (Stensrud and Eriksen, 2019). While international organisations have adopted the trope of environmental sustainability, in the PRC Ecological Civilisation has been at the centre. This poses a first epistemological and political problem. In the local semantic field, as Richard Edmonds (2011) has noted, the modern term for “environment”, *huanjing* 环境, is akin to the English word. It can refer to social spheres, such as the social environment (*shehui huanjing* 社会环境), but also to geographic ones, that is, natural environment (*ziran huanjing* 自然环境) or ecological environment (*shengtai huanjing* 生态环境). Its presence is widespread in the PRC, and one can easily find a sign calling to “protect the environment” (*baohu huanjing* 保护环境), indicating that one should not destroy the greens or litter. It differs from the Chinese concept of “ecology”, *shengtai* 生态, which, according to the authoritative Cihai Modern Dictionary of the Chinese Language (Cihai, 2022), refers more narrowly to living organisms (*shengwu* 生物). In contrast, its use in the English language is extended to larger interrelations between peoples, social groups, and even systems, with their environment (OED Online, 2022).

The way ecology is mobilised in the political discourse becomes clearer in the official catchphrase of China’s Ecological Civilisation: “clear water and green hills are indeed mountains of gold and silver” (*lüshui qingshan jiushi jinshan yinshan*绿水青山就是金山银山). It was during a visit to Yu Village in Anji, Zhejiang Province in 2005, when then Provincial Party Secretary, Xi Jinping, uttered these words. According to an editorial in the Party’s official newspaper, “ecological protection and economic development are not contradictory or mutually exclusive, but a dialectical and unified relationship” (Renmin Ribao, 2022). What has come down in history as the “Two Mountains Theory” initially referred to the success in decontaminating a mining area and its productive repurposing for tourism and bamboo plantations. More recently, President Xi Jinping has stated that “clean waters and green hills are natural and ecological wealth, and also social and economic wealth” (Renmin Ribao, 2022).

In this view, the ecology heralded in the Two Mountains Theory is both a bucolic and romanticised idea of “green nature” and a source of economic value. According to Duan Longlong and Wang Linmei (2020) the protection of “clear waters and green hills” includes two economic dimensions: ecological capital and environmental quality. The former refers to those resources that are prone to be exploited, such as mining, biodiversity, water, and forests, which, under the label of Ecological Civilisation, become economically relevant as agritourist destinations. The latter refers to any type of pollution that could affect the health and productivity of human resources.

Here is where the idea of Civilisation, or *wenming*, becomes relevant. In modern China, *wenming* is a normative, prescriptive, and evolutionist political device used to distinguish between stages of development.^3^ The aspiration to achieve the material and institutional foundations of *wenming* combines material aspects—that serve as benchmarks of a society’s progress—with the inherent “quality” (*suzhi* 素质) of the people. As a keyword of the Reform era (Kipnis, 2006), *suzhi* promotes ideas and practices of internal self-regulation and the acceptance of one’s social status. Those born into lower suzhi—e.g., due to their geographic, class, or ethnic origin—can cultivate it (*xiuyang* 修养) by means of education, nourishment, disciplining, training and/or labour (Anagnost, 2004; Jacka, 2009; Yan, 2003). In this light, the civilizing process implies taking task with raising the citizens’ “quality” (*suzhi*素质) to build a developed, modern and prosperous society (Lin, 2017: 10).

In political discourse, Ecological Civilisation (*shengtai wenming* 生态文明) corresponds to the fourth dimension of a series of Civilisational claims established by the Communist Party of China (CPC) since 1980. It follows Spiritual Civilisation (*jingshen wenming* 精神文明), Material Civilisation (*wuzhi wenming* 物质文明), and Political Civilisation (*zhengzhi wenming* 政治文明), each one focused on the ideological, economic, and legal stability of the country respectively (Geall and Ely, 2018: 1184; Dynon, 2008: 106). The implementation of these directives has varied in intensity, but they all remain part of the current political lexicon in the PRC. The particularity of *shengtai wenming*, however, is that it situates the CPC as acting through a continuum across stages of epoch-making contradictions that integrate the goals of modernisation with new ideological developments that legitimate the Party’s political position. In other words, it integrates the discourse of sustainability into the socio-political narratives of legitimacy, otherwise known as the Party’s historical governing mission (Holbig, 2009: 53; Hu et al., 2018: 12).

Through the practices of *suzhi*, *wenming* appears as an ideal of social organisation and a guide for human behaviour in the spiritual/ideological, material/economic, political, and environmental dimensions. In an epoch where the consequences of climate change become increasingly visible, delivering some form of ecological and material wellbeing adds to the CPC’s “performative governance”, both in its substantive and theatrical dimensions (Ding, 2020). Moreover, Ecological Civilisation entails an imaginary of socio-ecological harmony in the PRC, where market production and consumption continue to grow, and technology and science have solved the basic problems of pollution and environmental degradation (Hansen et al., 2018: 201). In this light, building an Ecological Civilisation is as much an environmental claim as a political project of moral legitimacy by the CPC (Ding, 2020; Dynon, 2008: 85).

The tensions present in a concept like Ecological Civilisation are not solely due to the way these concepts, ecology and civilisation, stand in relation to each other. In a context of sociogenic climate change, it is important to understand the role of Ecological Civilisation as an environmental narrative stemming from a particular political economy project, such as China’s socialist modernisation, and how it impacts in other landscapes via financial investments and infrastructure developments. The circulation of these narratives does not happen in a vacuum, and the prospects for disruptive possibilities are usually limited by the domestic and transnational power relations embedded in a globalised market economy.

## Ecological Civilisation meets new energy

Under Xi Jinping’s leadership, science and technology have played a central role in defining the Ecological Civilisation. The policy concept was first introduced by President Hu Jintao during the 17^th^ Congress of the Communist Party of China in 2007, being enshrined in the Party Constitution in 2012—the same year when Xi Jinping became Secretary General and President of the country—and 6 years later, in the national Constitution. Through the concept of Ecological Civilisation, the CPC has projected its legitimacy in historical and visionary terms (Li and Shapiro, 2020: 6), as the concept itself becomes a pathway for normative processes in the making (Geall and Ely, 2018).

The context in Chinese politics where the concept of Ecological Civilisation emerged is telling of a centralised effort to tackle the socioenvironmental consequences of almost three decades of accelerated economic development. Like other East Asian countries, the PRC adopted a mode of production that integrated their state-supported national industries with the global circulation of capital and goods. Between the 1960s and 1990s, Japan, South Korea, and Taiwan became manufacturing and innovation centres, while the cities of Hong Kong^4^ and Singapore established themselves as global financial and logistics hubs. These trajectories were closely observed by the reformist leaders in Beijing who, after the end of the Cultural Revolution and the death of Mao Zedong in 1976, started to envision plans to revitalise their socialist economy. Under the label of “reform and opening” (*gaige kaifang* 改革开放), the government ushered an era of modernisation and capital accumulation that liberated the productive forces, opened the country to investments, and empowered the markets over society (Arrighi, 2008; Wang, 2008).

Looking through the lens of political geoecology it is possible to observe how socio-political and socioenvironmental struggles produce sets of relations, domestically and transnationally, that are embedded in a globalised market economy. Since the 1980’s, the process of reform and opening has promoted deep transformations of the landscape and social life, positioning the PRC as an integral part of the capitalist world-system while bringing its citizens out of poverty.^5^ Starting along the coastal areas, once rural areas became industrial towns “churning out export commodities and, increasingly, producing for an expanding domestic market” (Chan et al., 2009: 331). The need to find new frontiers of growth has motivated the continuous transformation of China’s industrial output, moving from labour intensive to capital intensive industries, and currently, innovating in advanced and green technologies.

The extension and depth of these transformations are hard to grasp, but in just 40 years places like Shenzhen went from a cluster of plantations and fishing villages to a global megalopolis. Balancing politico-ideological premises with the necessity to grow and develop the economy, in 1979 the CPC set Special Economic Zones (SEZs, *jingji tequ* 经济特区) that acted as buffer regions experimenting with the market economy. A rural area known as Bao’an, located directly in the border with Hong Kong was rebranded as Shenzhen SEZ. The incentive of a disciplined, qualified, and low-cost labour force across the border was enough to mobilise industries and investments and turn Shenzhen into an industrial powerhouse (Vogel, 1989: 126). Today, Shenzhen hosts some of the most valuable and innovative companies in the country, such as Huawei, one of the world’s largest technology companies, and BYD, the world’s largest maker of NEVs, amongst many others (Nylander, 2017).

China’s reform has been enabled by the large consumption of fossil fuels. According to economist Li Minqi, China is the second largest oil consumer (after the United States) and accounted for 47% of the total growth of global oil consumption between 2000 and 2014 (2016: 143). The rapid wave of urbanisation increased the demand for energy-heavy materials, such as concrete and steel, and with larger infrastructures and more disposable income, Chinese roads started to accommodate increasingly more cars. By prioritising economic growth for more than 30 years, the PRC has become a global production and innovation centre, greatly improving the connectivity and infrastructure of the country (albeit with huge social and environmental consequences). Now, in an attempt to reach a higher stage of civilisational development, the country needs to overhaul its industrial capacity to reduce carbon emissions by adopting New Energies.

BYD has been one of the iconic companies spearheading China’s energy transition. Starting in 1995 as a manufacturer of rechargeable batteries competing with Japanese imports, the Build Your Dreams Corporation Limited became a central supplier of Li-ion batteries to Motorola and Nokia by the beginning of the 21^st^ century. In 2002 BYD was listed in the Hong Kong Exchange (HKEX), and the next year acquired Xi’an Tsinchuan Auto, which is now its automaking branch. The PRC government has heralded BYD as a national champion of the PRC’s industrial upgrade strategy known as “Made in China 2025” (*zhongguo zhizao* 2025 中国制造2025), for their focus on NEVs. BYD’s success in electromobility has also been noted by investors like Warren Buffet and secured them a partnership with German automaker Daimler. From a battery producer to an innovative company with worldwide presence, BYD cars and buses are a common sight in Chinese cities, where the electrification of public transportation is an integral part of building the Ecological Civilisation.

**Image 1.**
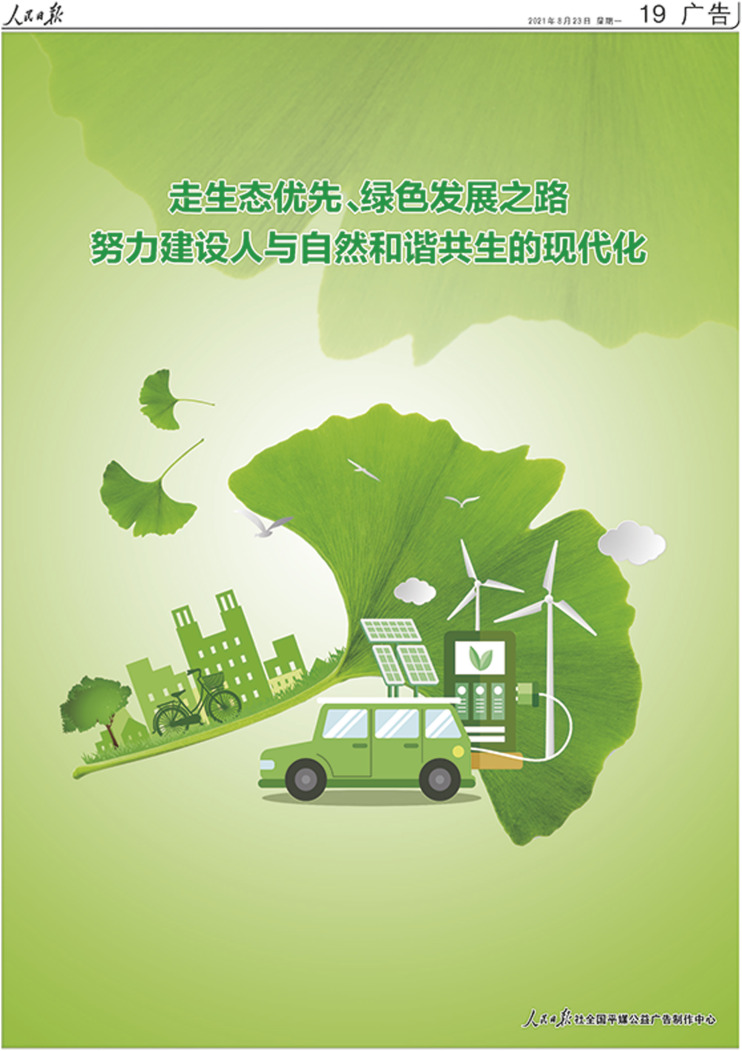
A promotional page from the People’s Daily from Monday, 23 August 2021. The caption reads “follow the path of ecological priority and green development and strive to build a modernization in which people and nature live together in harmony”.

Such combination of national industrial and ecological strategies is not casual, in fact, they are interwoven in the country’s overall economic roadmap to secure long-term growth (Image 1). “Made in China 2025” is a strategic plan issued by the Chinese government aimed at upgrading China’s manufacturing capabilities and transforming the country into a world-class manufacturing power by the year 2025. The plan focuses on 10 key sectors including advanced IT, robotics, aviation, and new energy vehicles. The goal is to increase the competitiveness of Chinese industries by upgrading technology and equipment, promoting innovation, and reducing the dependency on foreign technology.

In official documents it is stated that “with resources and environmental constraints increasing, costs for labour and of other factors of production rising, and the significant slowdown of investment and export growth, the reliance on a resource intensive development model cannot be sustained. We must accelerate the restructuring, transformation and upgrading of industrial quality and efficiency to strengthen economic growth and gain new advantages in international competition. The manufacturing industry is the focus, the challenge, and the solution” (State Council of the People’s Republic of China, 2015). Statements like this highlight the role that technological developments play in the conceptualisation of the Ecological Civilisation. However, together with proposing a national strategy to tackle anthropogenic climate change, these sets of policies aim at securing economic growth and development, albeit under the “green” label. This is the “New Development Philosophy” (*xin fazhan linian* 新发展理念), through which China pursues a perspective where “development must be innovative, coordinated, green, open, and shared, and accelerates the pace in creating a new development dynamic. […] China holds the view that clear waters and green mountains are invaluable assets, and that eco-environmental protection and improvement lead to greater productivity” (State Council of the People’s Republic of China, 2021: 4).

It is telling that the quality of industrial production and greater productivity stand at the centre of China’s ecological strategy, which renders the Ecological Civilisation as a development path coordinated with the tenets of the market economy. This brings us back to Tyfield’s thesis, that China’s electromobility transition is enmeshed by power relations that enhance state capacity. In this light, building China’s Ecological Civilisation implies the overhauling of its industrial base to become simultaneously green and profitable, putting pressure upon national and planetary ecosystems where the necessary raw materials are extracted and transformed (Li, 2016).

China’s Ecological Civilisation entails an environmental and industrial strategy with global reach. Its international projection is manifested in infrastructure projects—increasingly under the label of a “Green Belt and Road”—and through the flow of capital and resources that enable it. These engagements counterpose China’s economic might against the economic needs of developing countries, who set out strategies to maximize their chances of success capturing Chinese capital and market share (Labarca and Ampuero Ruiz, 2021). In this sense, the power relations established by Ecological Civilisation go beyond the notions of “environmental authoritarianism” within the Chinese polity, and project China’s dominance over the narratives and practices of sustainability through its engagements with other countries. In other words, by implementing technological responses to sociogenic climate change, such as the expansion of the NEV industry, China’s Ecological Civilisation triggers changes in other landscapes—like the expansion of mining activities—that are justified by discourses of green development.

## The power of new energy

By focusing on the NEV industry, Chinese companies have become strategically relevant in the economy of battery materials (such as lithium, cobalt, and graphite) and technologies. State subsidies to the production and consumption of NEVs has helped to position Chinese made cars domestically and globally. Four of the largest domestic automakers—SAIC Motor, BYD, Great Wall Motor, and Anhui Jianghuai Automobile Group (JAC)—ranked amongst the top 10 receivers of state subsidies (Kawase, 2022), which in some cases has been critical to achieve global market relevance. Such has been BYD’s case, that in the first half of 2022 surpassed Tesla as the world’s largest producer of electric vehicles by sales (Nuttall, 2022).

Cities in China and elsewhere have started to accommodate their infrastructure to facilitate the surge in demand for NEVs. The Chinese government set to build 4.8 million new chargers and 12,000 new charging and battery swapping stations available to the yet-to-be-met target of 5 million NEV drivers in 2020 (Ji and Huang, 2018). Centralised charging capabilities are still largely relegated to big cities in the country’s Eastern coast and are sparse along highways, which, in the words of a former editor-in-chief of the China Daily, makes NEVs a “city toy” (Kang, 2021).

**Image 2.**
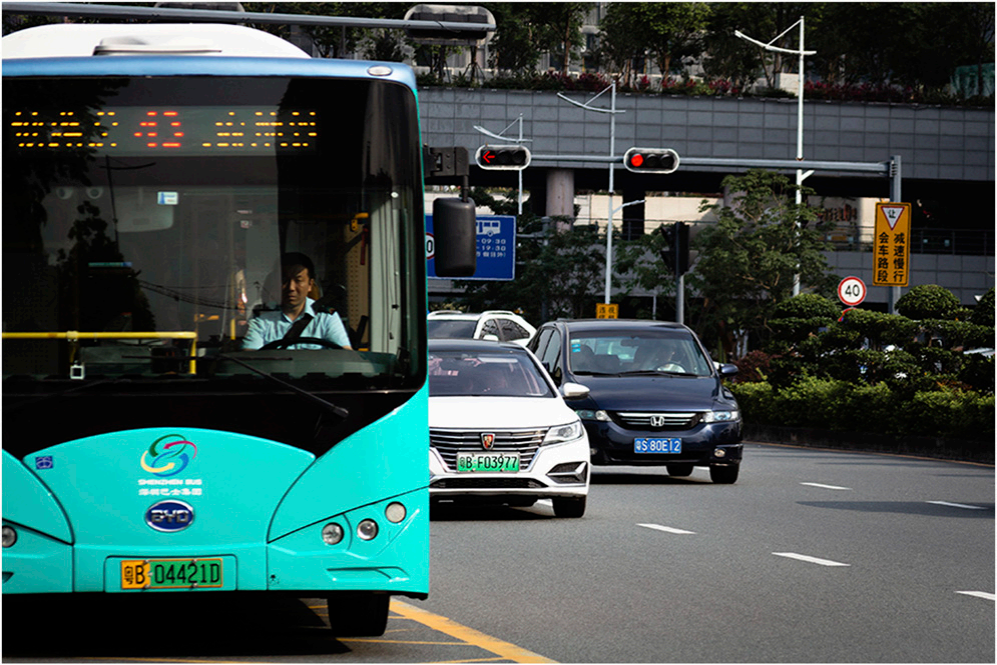
Different colours in the license plates differentiate NEVs from cars powered by fossil fuels. Image credit: Pablo Ampuero-Ruiz, 2019.

Despite these limitations, NEVs have become a desirable good as it relates to a low-carbon lifestyle. Among the most popular models in China, BYD’s Dolphin sells for €26,000,^6^ and the Hongguang Mini^7^ for less than €4000. But if the moral aspects of owning a NEV are not enough, in 2017 the Chinese government announced the nation-wide implementation of green license plates, which act as conspicuous markers that distinguish “green cars” from the blue plates of cars running on fossil fuels (Image 2). Applications for green license plates usually run smoother than for blue ones, although the wait can still extend for several years.

In the PRC, as elsewhere, New Energy technologies are usually phrased in moral terms, as benevolent “clean” and “green”, standing against “dirty” fossil fuels (Stensrud and Eriksen, 2019).^8^ While price is still a driving reason conditioning the adoption of NEVs, their additional moral dimension conceals critical junctions where, the imperatives of capital and sustainability, of old and new energies, appear as more synergetic and less transitional. Therefore, expanding on Tyfield’s notion of the “socio-technical regime” of e-mobility transition (2014: 590), I suggest that the Ecological Civilisation enacts a type of moral power that operates domestically but also internationally.

By exercising moral power, Chinese state and non-state actors justify the expansion of extraction, manufacturing, and consumption practices in a globalised market economy. They usually frame these actions as efforts to reduce emissions in the Chinese economy and are thus welcomed by the international community. But the pursuit for New Energy resources, such as lithium, cobalt, copper, or graphite, among many others, is happening amidst increasing competition between the United States, the European Union, and China. Looking through the lens of political geoecology, it is important to note that this competition has encouraged strategies of regionalisation of production chains, aiming at reducing dependency between competing countries. In 2021, the US Department of Energy released a plan to create a domestic battery supply chain, including mining, processing, and recycling (Williams, 2022). This has meant prioritising national-energy security in allowing Lithium Americas, a Canadian company, to extract lithium clay from Thacker Pass, northern Nevada, to the apprehension of environmental NGOs concerned about the threats to endemic biodiversity, local ranchers worried about the project’s intensive use of groundwater, and Paite and Shoshone tribal communities trying to defend sacred land, ancestral burial grounds, and their cultural subsistence (Rodeiro, 2022). In the European Union, a latecomer in the lithium battery competition, countries are looking to regionalise the value chain of lithium battery powered EVs. On the one hand, lithium extraction projects in Portugal and Spain are met with opposition by an increasingly elderly and impoverished local population, while, on the other, Germany hopes for an industrial revival of its eastern towns with a Tesla gigafactory and a lithium processing facility in Brandenburg, which adds to Volkswagen’s EV factory in Zwickau (Chazan and Miller, 2022).

In the PRC, the government has implemented consecutive plans to streamline its domestic NEV industry across all stages of production (State Council of the People’s Republic of China, 2020). This has motivated Chinese car and battery cell manufacturers to pursue increasing vertical integration of their supply chains. Once again, BYD has expanded their scope of investments by backing Chengxin Lithium Group, a lithium supplier with operations in China’s Sichuan province, Indonesia, and Argentina, but also by acquiring six mines in Africa (Lee, 2022; Wu, 2022). Another Chinese industrial juggernaut, CATL, the world’s largest Li-ion battery maker and supplier for Tesla and Volkswagen, has an extensive portfolio of investments and acquisition in mining operations for cobalt, copper, graphite, and lithium across the world (Bloomberg, 2022; Dempsey and Campbell, 2022). CATL’s might in the Li-ion battery economy is such that it has announced a €7.3 billion investment to build Europe’s largest battery plant in the small Hungarian town of Debrecen, with enough capacity to power 2 million cars per year (Dunai et al., 2022).

The pursuit for New Energy is fostering landscape transformations around the world. In many cases, there is resistance by the local population while national states welcome them as an opportunity to strengthen bilateral relations with the world’s most powerful economies. But from the perspective of these powerful economies, the control of access to New Energy resources is also a matter of sovereign power, relevant to their national security goals. In its quest to reduce carbon emissions without sacrificing its development goals, China has aimed at transforming its energy sources while sustaining and expanding its industrial capabilities. Chinese leaders see the energy transition as an opportunity to achieve energy and national security, pushing for increasing industrial vertical integration. This security focus is at the centre of the country’s strategy to cope with climate change between 2022 and 2035. For the Chinese government, it is important to develop the technologies and devices that support building an Ecological Civilisation to “carry out research on the assessment of the impact of climate change on food security, water security, *ecological security*, *transportation security*, *energy security*, and national defence security” (State Council of the People’s Republic of China, 2022; emphasis added).

With the imperative to control access to New Energy resources, China and other centres of the world economy foster the continuation of global relations of dependency between the sources of raw materials and the sources of capital. NEVs portend a new economic frontier for “green growth” in building the Ecological Civilisation, or paraphrasing from the book *China 2025*, it enables the protection of the environment *and* the productive forces (Hu et al., 2018: 108). In this formulation, the *ecological* stands as an economic opportunity connected to technological fixes, while the *civilisational* remains as a socio-technical regime with hierarchical power relations. In the lens of political geoecology, these considerations shed a new light on the celebration of NEVs as a “a win-win situation of technological progress and profit harvesting” (Qiu, 2016). First, because in the Ecological Civilisation the Party is ultimately who exercises the means of authoritarian environmentalism and sets the tone in the ongoing socio-political and socioenvironmental struggles. Second, because the realisation of environmental narratives like Ecological Civilisation through technological fixes makes it difficult to distinguish between state and corporate goals, particularly in the energy space, where these are often intertwined through projects of economic growth, state capacity, energy sovereignty, and geopolitical competition (cf. Blas and Farchy, 2021). Finally, because these intertwined political and economic relations of power enable disciplinary techniques that stifle the opposition against landscape transformations in China’s Shenzhen, Chile’s Atacama Desert or in Hungary’s Debrecen. In other words, the logic of Ecological Civilisation, as it is expressed through the transnational reach of the New Energy industries that enable it, mobilises forms of power that position New Energy investments and infrastructure as positive and desirable, furthering the reach of Chinese political and economic interests.

## Conclusion

To address the question of what type of New Energy does the Ecological Civilisation need, I have attempted at a reflection that unveils the semantics and the political geoecology of building an Ecological Civilisation. Stripped from its green mysticism, it is possible to observe that what drives the construction of an Ecological Civilisation is the growth imperative of the market economy, where New Energies appear as less of a transition and more of a continuation of the extraction, production, consumption, and discard practices inherent in the market economy.

China’s Ecological Civilisation emphasises the importance of technological developments to make human life more sustainable. But, as I have shown, it relies on a particular understanding of what that sustainability conveys, as it equates the ecological with a kind of biodiversity that is economically useful. Moreover, by mobilising the concept of *wenming* it stresses prescriptive aspects that position China’s Ecological Civilisation as a higher stage of (material) development. I showed how these limitations manifest themselves in the development of NEVs, a technology tasked with decontaminating the cities and reducing the consumption of fossil fuels. This socio-technical regime brings together technological and normative aspects, but also mobilises moral and sovereign forms of power that re-produce transnational dependencies around New Energy resources. Thinking through these critical junctions, I have problematised the idea of a New Energy for Ecological Civilisation by unveiling the tensions between the moralisation of New Energy, the dependencies around Chinese capital and industrial capacity, and the power relations underpinning the transformation of landscapes around the globe. In this constellation, New Energy operates as a constitutive part of a socio-technical regime where technologies of electromobility conflate normative and disciplinary mechanisms enacting an idea of Ecological Civilisation with national interests in a context of geopolitical and geoeconomic competition.

In closing this article, I want to emphasize the importance of critically examining the power relations underpinning environmental political discourses, policies, and practices. On the one hand, because there seems to be an implicit consensus about the role of technological fixes to address sociogenic climate change, without a clear understanding of the social, political, economic, and environmental challenges that they might entail. And, on the other, because efforts like building an Ecological Civilisation rely on domestic and transnational economic infrastructures operating in a very unequal world, and it is politically necessary to foreground those inequalities instead of taking them for granted. Finally, returning to China 2050, one should take note that building an Ecological Civilisation is an integral part of the Party’s historical project and its political legitimacy. In this light, I think that a deeper exploration of how these narratives become part of the collective imagination(s) and how they stand in front of other imaginations for green-er modernisations is urgently needed. Whose dream (or nightmare perhaps) are we inhabiting?

## Notes

1. All translations from Chinese are my own.

2. Establishing demonstration zones, where experimentation and adjustments can be performed before new measures are implemented in the whole country, is a recurrent aspect in Chinese policy making. In the case of building an Ecological Civilisation, a conceptualisation stage took place between 2005 and 2007, where researchers and policymakers discuss the definition and scope of the concept. Between 2007 and 2010, in the national framework stage, guidelines were produced for the building of city and county level experimentation zones. Then, between 2010 and 2019, the experimentation stage took place, starting with cities and counties in 12 provinces and expanding to the whole country Di et al. (2022).

3. It is important to note that colloquially speaking, the opposite of *wenming* is *luohou* 落后, usually translated as “backward” or “less developed.”

4. Hong Kong, under British control since 1841, was handed over to the PRC on 1 July 1997, with the promise of ‘one country, two systems’ (*yiguo liangzhi* 一国两制) until 2047.

5. Giovanni Arrighi (2008) provided a detailed analysis of the global re-emergence of China in the capitalist world-system. He argued in the lines of Joseph Stiglitz, that gradualism, as opposed to the ‘shock therapy’ implemented in the former Soviet bloc, explains the success of the Reform. This thesis finds new strength in Isabella Weber’s (2021) work on the economic debates among Party leaders in the 1980s. See Yu (2005) for more details about the politico-ideological tensions behind the Reform and the need to build a Spiritual Civilisation to contain the social effects of rapid economic development.

6. For reference, a comparable model in international markets, the 2023 Nissan Leaf, sell for €33,100 in the Netherlands, and a Tesla Model S starts at €51,990 through their official Web site.

7. Although SAIC-GM-Wuling sells this car exclusively in China, the model was considered the world’s best-selling electric vehicle of 2021.

8. Alexander Dunlap (2021) complicates this characterisation by arguing that many green energy technologies still rely on fossil fuels for their production and functioning. Therefore, he prefers the category of “fossil fuel+.”
